# Genetic history of the Koryaks and Evens of the Magadan region based on Y chromosome polymorphism data

**DOI:** 10.18699/vjgb-24-11

**Published:** 2024-02

**Authors:** B.A. Malyarchuk, M.V. Derenko

**Affiliations:** Institute of Biological Problems of the North of the Far-Eastern Branch of the Russian Academy of Sciences, Magadan, Russia; Institute of Biological Problems of the North of the Far-Eastern Branch of the Russian Academy of Sciences, Magadan, Russia

**Keywords:** Y chromosome, polymorphism, human populations, the Koryaks, the Evens, genetic history, Y-хромосома, полиморфизм, популяции человека, коряки, эвены, генетическая история

## Abstract

In order to clarify the history of gene pool formation of the indigenous populations of the Northern Priokhotye (the northern coast of the Sea of Okhotsk), Y-chromosome polymorphisms were studied in the Koryaks and Evens living in the Magadan region. The results of the study showed that the male gene pool of the Koryaks is represented by haplogroups C-B90-B91, N-B202, and Q-B143, which are also widespread in other peoples of Northeastern Siberia, mainly of Paleo-Asiatic origin. High frequency of haplogroup C-B80, typical of other Tungus-Manchurian peoples, is characteristic of the Evens of the Magadan region. The shared components of the gene pools of the Koryaks and Evens are haplogroups R-M17 and I-P37.2 inherited as a result of admixture with Eastern Europeans (mainly Russians). The high frequency of such Y chromosome haplogroups in the Koryaks (16.7 %) and Evens (37.8 %) is indicative of close interethnic contacts during the last centuries, and most probably especially during the Soviet period. The genetic contribution of the European males’ Y chromosome significantly prevails over that of maternally inherited mitochondrial DNA. The study of the Y chromosome haplogroup diversity has shown that only relatively young phylogenetic branches have been preserved in the Koryak gene pool. The age of the oldest component of the Koryak gene pool (haplogroup C-B90-B91) is estimated to be about 3.8 thousand years, the age of the younger haplogroups Q-B143 and N-B202 is about 2.8 and 2.4 thousand years, respectively. Haplogroups C-B90-B91 and N-B202 are Siberian in origin, and haplogroup Q-B143 was apparently inherited by the ancestors of the Koryaks and other Paleo-Asiatic peoples from the Paleo-Eskimos as a result of their migrations to Northeast Asia from the Americas. The analysis of microsatellite loci for haplogroup Q-B143 in the Eskimos of Greenland, Canada and Alaska as well as in the indigenous peoples of Northeastern Siberia showed a decrease in genetic diversity from east to west, pointing to the direction of distribution of the Paleo-Eskimo genetic component in the circumpolar region of America and Asia. At the same time, the Evens appeared in the Northern Priokhotye much later (in the XVII century) as a result of the expansion of the Tungusic tribes, which is confirmed by the results of the analysis of haplogroup C-B80 polymorphisms

## Abstract (ru)

Для прояснения истории формирования генофондов коренного населения Северного Приохотья изучен полиморфизм Y-хромосомы у коряков и эвенов Магаданской области. Результаты исследования показали, что мужской генофонд коряков представлен гаплогруппами C-B90-B91, N-B202, Q-B143, распространенными также у других народов Северо-Востока Сибири преимущественно палеоазиатского происхождения. Для эвенов Магаданской области характерна высокая частота гаплогруппы C-B80, свойственной и для других тунгусо-маньчжурских народов. Общим компонентом генофондов коряков и эвенов являются гаплогруппы R-M17 и I-P37.2, унаследованные в результате метисации с пришлым восточноевропейским (преимущественно русским) населением. Высокая частота такого рода гаплогрупп Y-хромосомы у коряков (16.7 %) и эвенов (37.8 %) свидетельствует об интенсивных межэтнических контактах на протяжении последних столетий, и особенно, по всей видимости, в советское время. Причем генетический вклад со стороны европейских мужчин (по Y-хромосоме) существенно преобладает над таковым со стороны женщин (по митохондриальной ДНК). Исследование разнообразия гаплогрупп Y-хромосомы показало, что в генофонде коряков сохранились только относительно молодые филогенетические ветви. Возраст наиболее древнего компонента генофонда коряков (гаплогруппа C-B90-B91) оценивается примерно в 3.8 тыс. лет, возраст более молодых гаплогрупп Q-B143 и N-B202 составляет примерно 2.8 и 2.4 тыс. лет соответственно. Гаплогруппы C-B90-B91 и N-B202 являются сибирскими по происхождению, а гаплогруппа Q-B143, вероятно, унаследована предками коряков (и других палеоазиатских народов) от палеоэскимосов в результате их миграций на Северо-Восток Азии из Америки. Анализ микросателлитных локусов для гаплогруппы Q-B143 у эскимосов Гренландии, Канады и Аляски, а также у представителей коренного населения Северо-Востока Сибири выявил снижение генетического разнообразия с востока на запад, что указывает на направление распространения палеоэскимосского генетического компонента в циркумполярном регионе Америки и Азии. Эвены же появились в Северном Приохотье намного позже (в XVII в.) в результате экспансии тунгусских племен, что подтверждается данными анализа полиморфизма гаплогруппы C-B80.

## Introduction

The extreme Northeast of Siberia is inhabited by the Chukotka- Kamchatkan peoples (the Chukchis, Koryaks, Itelmens) and the Eskimos, which are characterized by genetic peculiarities and occupy a distinct position among the ethnogeographical groups of Northern Eurasia (Rasmussen et al., 2010; Fedorova et al., 2013; Cardona et al., 2014; Pagani et al., 2016; Pugach et al., 2016; Gorin et al., 2022). According to paleogenomic data, the genetic specificity of these peoples is due to their ancient Paleo-Siberian genetic substrate, inherited in part by the Native Americans (Sikora et al., 2019). Meanwhile, the results of the analysis of autosomal loci polymorphism in the indigenous Siberian populations have shown that in the east of Siberia the appearance of alleles of European origin is estimated to be relatively recent (about 3–6 generations ago), which is associated with the Russian discovery of Siberia, starting mainly from the XVII century and especially intensive during the Soviet period (Cardona et al., 2014). Moreover, various studies demonstrate that the flow of European genes into the gene pools of the indigenous populations of Northeastern Siberia was carried out predominantly by men (Balanovska et al., 2020a, b; Agdzhoyan et al., 2021; Solovyev et al., 2023). In this regard, the contribution of European Y chromosome variants to the gene pools of indigenous peoples of Northeastern Siberia and other Arctic regions usually exceeds that of European maternally inherited mitochondrial DNA (mtDNA) variants (Bosch et al., 2003; Rubicz et al., 2010; Dulik et al., 2012; Olofsson et al., 2015).

The results of genetic studies of the indigenous populations of the northern coast of the Sea of Okhotsk – the Koryaks and Evens of the Magadan region – have shown that they have a very low frequency of European mtDNA variants (only in the Evens it reaches 4 %) (Derenko et al., 2023), and according to the results of genome-wide analysis, the frequency of the European genetic component in the Northeastern Siberian populations has significantly increased only in the last ~100 years (Cardona et al., 2014). Most likely, this may be related to the increased European contribution by males, and therefore, the aim of this paper is to analyze the Y chromosome polymorphism in the indigenous populations of the Magadan region.

## Materials and methods

Unrelated males from the indigenous populations of the Magadan region (the Koryaks and Evens) were studied (Supplementary Materials 1 and 2)1. Based on survey data, the Koryaks (N = 36) and Evens (N = 61) studied had identified themselves as belonging to the above ethnic groups for at least 2–3 generations. According to the results of mtDNA analysis, all individuals studied are characterized by haplotypes of Northeast Asian origin.


Supplementary Materials are available in the online version of the paper: https://vavilov.elpub.ru/jour/manager/files/Suppl_Malyar_Engl_28_1.pdf


DNA was extracted and purified from whole blood as we previously described (Derenko, Malyarchuk, 2010). Samples were genotyped for 12 microsatellite (STR) loci (DYS19, DYS385a, DYS385b, DYS389I, DYS389II, DYS390, DYS391, DYS392, DYS393, DYS437, DYS438, DYS439) using PowerPlex Y System (Promega Corporation, Madison, WI, USA). Alleles were detected by capillary electrophoresis on ABI 3500xL Genetic Analyzer (Applied Biosystems, USA). The results were analyzed using the programs Genscan v. 3.7 and Genotyper v. 3.7 (Applied Biosystems). Data for DYS385 loci were not considered in the statistical analysis because the order of the DYS385a and DYS385b loci on the Y chromosome is unknown. The number of repeats at the DYS389II locus was determined by subtracting the length of the smaller repeat (DYS389I) from the length of the larger repeat (DYS389II).

Y chromosome haplogroups were determined by direct DNA sequencing or restriction fragment length polymorphism analysis of haplogroup markers as we described previously (Malyarchuk et al., 2013). Data on variability of the B77, B79, B80, B81, B90, B91, B92, B94, B143, B186, B202, B203, B204, and B471 loci were obtained earlier in studies of whole Y chromosome variability in different ethnic groups, including some Koryak and Even individuals from the Magadan region (Karmin et al., 2015).

The Vp statistic, the average dispersion of the number of repeats in STR loci, was used to estimate intrapopulation genetic diversity (Kayser et al., 2001). The evolutionary age of the Y chromosome haplogroups was calculated based on the analysis of the average number of repeats in loci and their variance (Zhivotovsky et al., 2004). The mutation rate value used in the calculations, 2.79·10–3 substitutions per locus per generation, was obtained by averaging mutation rates for the 10 Y chromosome loci analyzed, according to Ballantyne et al. (2010). The program Network 10.2 (www.fluxus-engineering. com) was used to construct median networks of the Y chromosome STR haplotypes.

## Results and discussion

The results of the study of Y chromosome polymorphism showed that the male gene pool of the Koryaks living in the Magadan region is represented mainly by haplogroups C, N, and Q (Table 1). European lineages in the Koryaks were found at a frequency of 16.7 % for haplogroups R-M17, I-M253, and I-P37.2. The frequency of European haplogroups is even higher among the Evens – 37.8 %. They are represented by haplogroups R-M17, R-M269, I-P37.2, as well as N-B186, which is characteristic of the peoples of Northeastern Europe (Karmin et al., 2015). The East Asian component of the Even gene pool consists of various subgroups of haplogroup C (55.7 % in total). In addition, haplotypes belonging to haplogroup Q-M3, which is widespread among the Native Americans and Eskimos, have been found in the Evens.

**Table 1.**
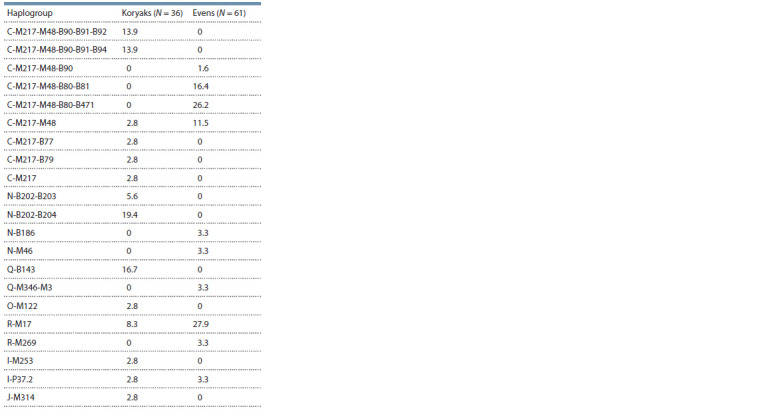
Frequency (in %) of Y chromosome haplogroups in the Koryaks and Evens of the Magadan region

Haplogroup C variants in the Koryak and Even populations differ significantly. The Koryaks are characterized by the B90 and B91 specific markers, while the Evens fall into the B80- defined subgroup. According to the results of whole-genome studies, the B90 marker is specific for the Y chromosomes of the indigenous populations of Northeastern Siberia (the Koryaks, Evenks, and Ulchi) (Karmin et al., 2015; Balanovska et al., 2018), and the B91-defined subgroup is present only in the Koryaks (Karmin et al., 2015). Its frequency in the Koryaks of the Magadan region is 27.8 % (see Table 1).

According to the results of molecular dating based on the analysis of polymorphism associated with single nucleotide substitutions (SNP) in whole Y chromosomes, the age of the B91 subgroup is estimated at 3.8 (3.0–4.7) thousand years (Karmin et al., 2015). The age of the upstream C-B90 subgroup is approximately 5.0 (4.2–5.7) thousand years. Based on the similarity of STR profiles, B90 haplotypes appear to be predominantly distributed in Northeastern Siberia, since, in addition to the Koryaks, Evenks and Ulchi, homologous STR haplotypes are observed in the Yakuts, Yukaghirs, Itelmens, and Evenks (Adamov D.S. Summary table of Y-STR haplotypes of haplogroup C-M48 of Yakut-Sakha. 2019. https://www.researchgate.net/profile/Dmitry-Adamov/ publications/ (Reference date: September 5, 2023)). In our study, a single homologous B90 haplotype (similar to that of the Koryaks) was also found in the Evens

In the Evens, the C subgroup, marked by a substitution at locus B80, is mainly distributed (see Table 1). It is known that B80 haplotypes, in addition to the Evens, are also characteristic of other Tungus-Manchurian peoples (the Orochens, Evenks, and Manchurians) (Yu et al., 2023). The evolutionary age of this subgroup, according to the SNP data, is 1.7 (1.2– 2.2) thousand years (Karmin et al., 2015). The results of the analysis by H.-X. Yu et al. (2023) have shown that the age of the B80 subgroup is estimated to be about 2 thousand years, while the B81 and B471 haplotypes specific to the Evens originated in the Amur region and spread to Northeastern Siberia as a result of the migrations of the Tungus ancestors in the last approximately 1.5 thousand years.

The N haplogroup in the Koryaks of the Magadan region is represented exclusively by the N-B202 branch (25 %). The same subgroup predominates in the gene pool of the Chukchi (Karmin et al., 2015; Ilumäe et al., 2016; Agdzhoyan et al., 2021), and is also found in the neighboring peoples – the Itelmens and Eskimos (Agdzhoyan et al., 2021). The age of the N-B202 branch is approximately 2.4 (1.8–3.1) thousand years (Ilumäe et al., 2016). This haplogroup consists of two subgroups, the older N-B204 (estimated to be about 1.4 thousand years old based on STR haplotype diversity) and the younger N-B203 (about 600 years old) (Agdzhoyan et al., 2021). In the Chukchi, both subgroups are present to a nearly equal extent, with the older subgroup N-B204 predominating in the Koryaks (see Supplementary Material 1). In the Evens of the Magadan region, haplogroup N was found at a relatively low frequency (6.6 %) and is represented by different haplotypes. In this respect, the Magadan Evens are similar to the Kamchatkan Evens, but differ from the Okhotsk Evens, who are characterized by the “Amur region” subgroup N-B479 at a frequency of 10 % (Agdzhoyan et al., 2019).

Haplogroup Q represents the oldest component of the gene pools of the indigenous populations of Siberia and America. Haplogroup Q-F903 was found in an Upper Paleolithic inhabitant of Eastern Siberia (the Afontova Gora archaeological site, approximately 17 thousand years old) (Raghavan et al., 2014), and haplogroup Q-B143 was revealed in Northeastern Siberia (the Duvanniy Yar site, about 10 thousand years old) (Sikora et al., 2019). The same haplogroup was reported in a representative of the Paleo-Eskimo Sakkak culture who lived in Greenland about 4 thousand years ago (Rasmussen et al., 2010). Currently, haplogroup Q-B143 is distributed only among the indigenous populations of the American Far North, Greenland and Siberia (Malyarchuk et al., 2011; Karmin et al., 2015; Grugni et al., 2019; Luis et al., 2023). In the Koryaks of the Magadan region, this Q haplogroup was detected with a frequency of 16.7 % (see Table 1). According to indirect data (based on the frequencies of haplogroups Q(xM346) and Q-NWT01, as well as on the similarity of STR haplotypes), haplogroup Q-B143 is present in the Koryaks of Kamchatka (with frequency varying from 6 to 18 %)3 (Karafet et al., 2018), in the Chukchi (13 %) (Kharkov V.N. Structure and phylogeography of the gene pool of the indigenous population of Siberia according to Y chromosome markers. Dr. Biol. Sci. Diss. Tomsk, 2012. (in Russian)), in the Yukaghirs (30.8 %) (Pakendorf et al., 2006), and it has also been found with high frequencies (up to 50 %) in the Eskimos of Alaska, Canada and Greenland (Dulik et al., 2012; Olofsson et al., 2015; Luis et al., 2023).

The presence of haplogroup Q-B143 in the Northeast of Siberia about 10 thousand years ago and at present suggests that Q-B143 is the most ancient Siberian component that has been a part of the gene pools of the Paleo-Asiatic peoples and their ancestors. Archaeological data, as well as the results of the study of haplogroup Q polymorphism, showed that about 5 thousand years ago the carriers of haplogroup Q-B143 (as well as unsuccessful Q-L713 and Q-preM120) migrated from Siberia to America and then to Greenland and became the founders of the Paleo-Eskimo culture (Grugni et al., 2019). However, the results of the Q-B143 dating showed that the age of this haplogroup in modern Koryaks is only about 2.8 thousand years, which indicates the possibility of back migration of the carriers of these haplotypes (most likely, the Paleo-Eskimos) from North America to Northeast Asia (Grugni et al., 2019). Similarly, the results of studies of STR variability within haplogroup Q-B143 in Greenlandic and North American Eskimos showed that the diversity and evolutionary age of haplotypes in Greenlandic Eskimos are higher than in Canadian and Alaskan Eskimos (Olofsson et al., 2015; Luis et al., 2023). In this connection, these authors suggested that haplogroup Q-B143 was spread by the Paleo- Eskimos from the east to the west of America and, moreover, became one of the main components of the gene pool of the Neo-Eskimos, which most likely formed in the north of America about 700 years ago.

Since Luis et al. (2023) did not investigate Q-B143 haplotypes in the indigenous populations of Northeast Asia, we analyzed STR haplotype diversity in samples of Greenlandic, Canadian, and Alaskan Eskimos (based on data from Dulik et al. (2012), Olofsson et al. (2015), Luis et al. (2023)), and in the Koryaks, Yukaghirs, and Chukotkan Eskimos (according to Pakendorf et al. (2006), Luis et al. (2023), and the present study). The results of our study showed that, indeed, Northeast Asian sample has the lowest diversity of Q-B143 haplotypes compared to Greenlandic and North American ones, indicating that these haplotypes appeared in Northeast Asia later than in North America and Greenland (Table 2).

**Table 2.**
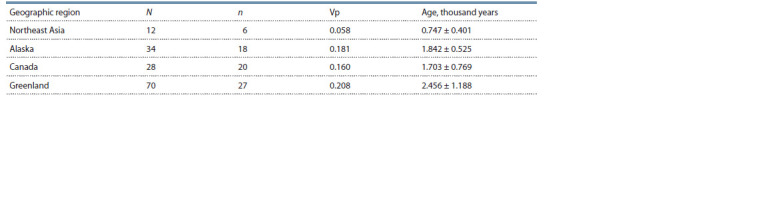
Diversity and evolutionary age of the Q-B143 STR haplotypes in the Eskimo and Paleo-Asiatic peoples N is the sample size, n is the number of STR haplotypes, Vp is the variance of the number of repeats in STR loci.

It is necessary to note the discrepancy between the dates obtained using STR markers and whole-genome SNP data, because the evolutionary age of haplogroup Q-B143 in the Koryaks according to SNP data (2.8 ± 0.9 thousand years as per Grugni et al. (2019)) exceeds that obtained using STR markers for the indigenous population of Northeastern Siberia (0.7 ± 0.4 thousand years) (see Table 2). This is most likely due to the very large mismatch in the number of variable positions for the compared genetic systems, the high probability of recurrent (forward and reverse) mutations for rapidly evolving STR loci, and the dependence of such mutational events on the age of haplogroups. Therefore, it is likely that STR dates close to the whole-genome ones can be obtained only for young branches (Agdzhoyan et al., 2021). Thus, if we focus on the whole-genome SNP dating (as more accurate), we can assume that the appearance of haplogroup Q-B143 in Northeast Asia occurred long before the appearance of the Neo-Eskimos and is thus associated with the migrations of the Paleo-Eskimos. The possibility of such events is evidenced by archaeological data, according to which the Paleo-Eskimo cultural tradition was established in Chukotka about 3.0–3.5 thousand years ago (the Chertov Ovrag site on Wrangel Island and the Unenen settlement), as well as in the Sea of Okhotsk’s northern coasts by representatives of the Tokarev culture (probable ancestors of the Koryaks) about 2.8 thousand years ago (Grebenyuk et al., 2019). The low level of diversity of Northeastern Siberian STR haplotypes and their peripheral position in the median network among the huge number of Q-B143 haplotypes of Arctic peoples indicate a very small number of successful (in terms of reproduction) migrations of the Paleo-Eskimos to the Asian coast (see the Figure). In fact, a single haplotype (ht20 in the Figure) is the most likely ancestor for the other haplotypes identified in the Koryaks and Yukaghirs.

**Fig. 1.**
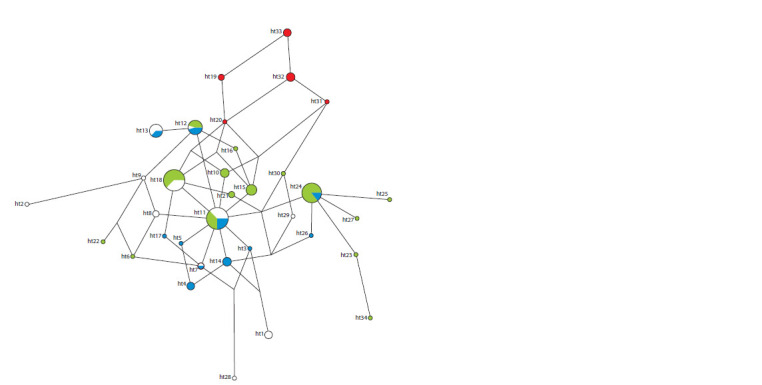
Median network of STR haplotypes belonging to Y chromosome haplogroup Q-B143 in the Eskimos of Greenland (green), Canada (blue), Alaska (white) and in the indigenous population of Northeast Siberia (the Koryaks, Yukaghirs, Eskimos) (red).

The low level of heterogeneity of Q-B143 haplotypes in the indigenous populations of Northeastern Siberia also indicates that the most ancient haplotypes, ancestral to the haplotypes of the Paleo-Eskimos of the north of America and Greenland have not been preserved in their gene pools. This seems quite likely, given the low effective population size of Northeastern Siberians and the increasing role of genetic drift under these conditions, as well as the continuing influence from neighboring Siberian populations. It is known that periods of almost complete population replacements occurred more than once during the 35 thousand years of Siberia’s population history (Sikora et al., 2019).

Traces of later contacts between the Neo-Eskimos and Paleo- Asiatic peoples are very strongly recognized by genetic data. The Neo-Eskimos were formed on the basis of two genetic components – the Paleo-Eskimo and the Paleo-Indian ones (Flegontov et al., 2019; Sikora et al., 2019). At that, the Paleo-Indian component of the Neo-Eskimos is well recognized by mtDNA haplogroups (A2a, A2b) and Y chromosome haplogroups (Q-M3). Therefore, by the presence of these haplogroups, it is possible to estimate the genetic contribution of the Neo-Eskimos. Based on mtDNA markers, the frequency of haplogroups A2a and A2b is very high in the Asian Eskimos and Chukchi, while among other Paleo-Asiatic peoples, these haplogroups were found only in the Koryaks at frequencies ranging from 2.7 to 9.1 %(Starikovskaya E.B. Phylogeography of the mitogenomes of indigenous populations of Siberia. Dr. Biol. Sci. Diss. Novosibirsk, 2016. (in Russian)). (Derenko et al., 2023). On the Y chromosome, the Paleo-Indian contribution, marked by haplogroup Q-M3, in the Chukchi and Kamchatkan Koryaks has been estimated to be 11.0 and 6.1 %, respectively(Kharkov V.N. Structure and phylogeography of the gene pool of the indigenous population of Siberia according to Y chromosome markers. Dr. Biol. Sci. Diss. Tomsk, 2012. (in Russian)). The Q-M3 haplogroup was not detected in the Koryak population we studied; however, the frequency of this haplogroup in the Evens is 3.3 % (see Table 1). The most probable reason for the appearance of the “American” haplogroup Q-M3 in the Evens of the Magadan region is interethnic contacts, either with the Koryaks or directly with the Eskimos or related tribes, which, according to archaeological, ethnographic and linguistic data, could have lived on the Sea of Okhotsk coast as early as in the beginning of the 2nd millennium AD (Burykin, 2001).

The high level of interethnic admixture in Northeastern Siberia, mentioned in a number of studies (Khakhovskaya, 2003; Balanovska et al., 2020a, b), is associated with the economic development of this region, first by Russian explorers and then, in the Soviet period, by numerous migrants, mainly of Eastern European origin. In the present study, we also found a high frequency of Y chromosome haplogroups characteristic of Eastern Europeans (and Russians, in particular): haplogroups R, I and J (Derenko et al., 2006; Balanovsky et al., 2008). In the Koryaks, their frequency was 16.7 %, and 37.8 % in the Evens (see Table 1). Moreover, in the Evens, the diversity of R-M17-haplotypes significantly exceeds that of the C-M217 haplogroup characteristic of the Evens themselves (Vp = 0.225 and 0.1, respectively). Meanwhile, the results of the study of maternally inherited mtDNA variability in the Koryaks and Evens of the Magadan region showed that they have a very low frequency of European mtDNA variants (up to 4 % in the Evens) (Derenko et al., 2023). The obtained results, thus, testify to a long history of admixture between the indigenous and immigrant populations in the territory of the Magadan region, as well as to the fact that immigrant men were predominantly involved in interethnic marriages and most of the children of such marriages were likely to be registered as indigenous, which is also typical of other areas of Northeastern Siberia according to demographic data (Khakhovskaya, 2003; Balanovska et al., 2020b).

## Conclusion

The results of the study have shown that the male gene pools of the indigenous populations of the Magadan region – the Koryaks and Evens – differ significantly in their structure. The Koryaks have a specific set of Y chromosome haplogroups similar to those of the indigenous peoples of Northeastern Siberia: C-B90-B91, N-B202, Q-B143, while the Evens are characterized by a high frequency of haplogroup C-B80, common among the Tungus-Manchurian peoples. The haplogroups common to the Koryaks and Evens (such as R-M17 and I-P37.2) were obtained from Eastern European migrants as a result of interethnic admixture. The high frequency of this kind of Y chromosome haplogroups in the indigenous peoples of the Magadan region testifies to rather intensive interethnic contacts, mainly from the side of Eastern European males. The analysis of the evolutionary age of aboriginal Y chromosome haplogroups has shown that the gene pools of the Koryaks and Evens are represented by relatively young phylogenetic branches. In the Koryaks, the age of the oldest component of the gene pool (haplogroup C-B91) is estimated to be about 3.8 thousand years; later, haplogroups Q-B143 (about 2.8 thousand years ago) and N-B202 (about 2.4 thousand years ago) appeared in the Koryak gene pool. The Q-B143 haplogroup was most likely inherited by the ancestors of the Koryaks (as well as other Paleo-Asiatic peoples) from the Paleo-Eskimos as a result of their migrations along the Sea of Okhotsk coast. The Evens appeared in the Northern Priokhotye much later (in the XVII century) as a result of the expansion of Tungusic-speaking populations, which is confirmed by the results of the analysis of haplogroup C-B80 polymorphism.

## Conflict of interest

The authors declare no conflict of interest.
